# Guanidinium Chloride-Induced Haemolysis Assay to Measure New Permeation Pathway Functionality in Rodent Malaria *Plasmodium berghei*

**DOI:** 10.3390/biom14070781

**Published:** 2024-06-30

**Authors:** Mitchell L. Trickey, Natalie A. Counihan, Joyanta K. Modak, Tania F. de Koning-Ward

**Affiliations:** 1 School of Medicine, Deakin University, Geelong 3216, Australia; mtrickey@deakin.edu.au (M.L.T.); n.counihan@deakin.edu.au (N.A.C.); joyanta.modak@deakin.edu.au (J.K.M.); 2 Institute for Mental and Physical Health and Clinical Translation (IMPACT), Deakin University, Geelong 3216, Australia

**Keywords:** *Plasmodium berghei*, new permeation pathway, *Plasmodium* surface anion channel, osmotic lysis, guanidinium chloride

## Abstract

Parasite-derived new permeation pathways (NPPs) expressed at the red blood cell (RBC) membrane enable *Plasmodium* parasites to take up nutrients from the plasma to facilitate their survival. Thus, NPPs represent a potential novel therapeutic target for malaria. The putative channel component of the NPP in the human malaria parasite *P. falciparum* is encoded by mutually exclusively expressed *clag3.1/3.2* genes. Complicating the study of the essentiality of these genes to the NPP is the addition of three *clag* paralogs whose contribution to the *P. falciparum* channel is uncertain. Rodent malaria *P. berghei* contains only two *clag* genes, and thus studies of *P. berghei clag* genes could significantly aid in dissecting their overall contribution to NPP activity. Previous methods for determining NPP activity in a rodent model have utilised flux-based assays of radioisotope-labelled substrates or patch clamping. This study aimed to ratify a streamlined haemolysis assay capable of assessing the functionality of *P. berghei* NPPs. Several isotonic lysis solutions were tested for their ability to preferentially lyse infected RBCs (iRBCs), leaving uninfected RBCs (uRBCs) intact. The osmotic lysis assay was optimised and validated in the presence of NPP inhibitors to demonstrate the uptake of the lysis solution via the NPPs. Guanidinium chloride proved to be the most efficient reagent to use in an osmotic lysis assay to establish NPP functionality. Furthermore, following treatment with guanidinium chloride, ring-stage parasites could develop into trophozoites and schizonts, potentially enabling use of guanidinium chloride for parasite synchronisation. This haemolysis assay will be useful for further investigation of NPPs in *P. berghei* and could assist in validating its protein constituents.

## 1. Introduction

Global gains that have been made in combatting malaria are at risk due to *Plasmodium* parasites acquiring resistance to frontline anti-malarial drugs. Worryingly, in parts of Southeast Asia and in several regions in Africa, there is delayed clearance of parasites following artemisinin combination therapies (ACTs) [1,2,3]. This will likely result in further severe infections and deaths, particularly in children under the age of five, who are most at risk of infection. The discovery of novel drug targets is therefore critical in the fight against malaria.

An increase in the prevalence of parasite drug resistance has driven research to investigate novel drug targets unique to the *Plasmodium* parasite, which are essential for their survival. One such candidate is the new permeation pathways (NPPs) on the red blood cell (RBC) surface that have been implicated in nutrient transport [4]. NPPs are parasite-derived and have been widely studied in the human malaria species *P. falciparum* with respect to transport properties. NPPs have been shown to facilitate the transport of low molecular weight solutes such as sugars and sugar alcohols, amino acids (including isoleucine), small peptides, some vitamins (including pantothenate), purines and ions [5]. The selective inhibition of NPPs using the anion transport blocker furosemide arrests cell development and leads to parasite death, lending further support to the NPPs being a potential drug target [6]. Despite the increased permeability of iRBCs being discovered in 1948 [7], followed by the first description of NPPs [8] and the subsequent detection of an induced channel [9], the exact composition of the channel(s) is still unknown. Consequently, substantial questions remain regarding the molecular make-up of NPPs that warrant further research. NPPs have characteristics of an anion selective channel; hence, it is also referred to as the *Plasmodial* surface anion channel (PSAC) [9]. There has been rigorous debate regarding whether the channel is in fact made up of one or several proteins and whether it is either parasite encoded or host derived [10]. The RhopH protein complex, comprising three proteins, RhopH1, RhopH2 and RhopH3, has been linked to NPP functionality in *P. falciparum*. RhopH1, encoded by two mutually exclusively expressed genes, *clag3.1* and *clag3.2*, was the first molecular component linked to the NPP [11]. RhopH1 *clag3.1/3.2* contains a single transmembrane domain (TMD) and hypervariable region (HVR) that, by proteolytic susceptibility assays, was revealed to be exposed at the RBC surface. RhopH1 is, therefore, the putative channel component of the NPP [12]. However, questions remain regarding the contribution of RhopH1 to the NPP channel, since RhopH1 *clag3.1/3.2* may not be essential to survival [13]. Studies have revealed that *clag3.1/3.2* genes can be knocked out without killing parasites, exhibiting only a delayed growth phenotype when grown in modified media that resemble human plasma [13,14,15]. In contrast, conditional knockdown or knockout of *rhoph2* and *rhoph3* leads to loss of NPP function and parasite death [16,17,18]. The soluble form of the RhopH complex has since been solved, revealing that the TMD helices in the RhopH complex are shielded during transport, and as a consequence, significant conformational change would need to occur for insertion of the complex into the host membrane [19,20]. There remains the possibility that another essential protein interacts with the RhopH complex and forms the channel, but RhopH2 immunoprecipitation experiments have failed to identify any such proteins, of either parasite or host origin [16].

A significant challenge in deciphering the role of *P. falciparum* RhopH1 in NPPs is the presence of five *clag* paralogs that encode this protein, potentially explaining why *clag3.1/3.2* has been shown to be dispensable [21,22]. Unfortunately, simultaneous knockout of all *clag* genes to determine essentiality is technically unfeasible in *P. falciparum*. The RhopH complex in *P. berghei*, on the other hand, contains only two paralogs of the *clag* gene, and this species is more genetically tractable compared to *P. falciparum* [23,24]. Therefore, *P. berghei* may provide a better model to reveal the contribution of *clag* genes to NPPs. Generation of *clag* gene knockouts in *P. berghei*, followed by investigation of NPP functionality, could directly reveal *clag* essentiality to the NPP.

It has been established that parasitised rodent RBCs also have an increased permeability to a number of organic compounds. *P. berghei* iRBCs have been shown to be permeable to labelled L-glucose [25], and patch clamping has been used to quantify increases in permeability [26]. *P. vinckei* iRBCs have been shown to exhibit an increased influx of choline that could be inhibited by furosemide [27]. Similarly, *P. yoelii* induced the permeation of iRBCs to two enantiomers of adenosine, which could also be inhibited by furosemide [28]. There is, therefore, substantial evidence for the use of solute uptake as an indicator of NPP functionality in rodent models of malaria.

A large range of solutes have been used in combination with transport techniques, including patch-clamp studies, to assess NPP activity across *Plasmodium* spp. [9,26]. Of particular interest to this study, sorbitol osmotic lysis assays have been an important tool for assessing the activity of NPPs in *P. falciparum* parasites and, based on its selective lysis of RBCs infected with trophozoite and schizont stage parasites, for parasite synchronisation [29]. It is therefore conceivable that an NPP-specific osmotic lysis assay measuring haemolysis would aid NPP studies in *P. berghei*. However, physiological differences exist between rodent and human RBCs [30,31,32]; consequently, an osmotic lysis assay must be tailored for *P. berghei* such that the lysis solution specifically enters iRBCs and is not taken up by endogenous channels.

The aim of the present research was to find an appropriate isotonic solution that is capable of selectively lysing *P. berghei* parasitised rodent RBCs by measuring the degree of haemolysis and to show that import occurs via NPPs. Several isotonic solutions were tested for their ability to preferentially cause lysis of iRBCs compared to uRBCs and their consequential effect on parasite development. Guanidinium chloride and L-alanine solutions were both found to lyse iRBCs, with guanidinium proving to be more effective. The pathway of guanidinium-induced lysis was shown to be specifically via NPPs, and guanidium did not lyse early ring-stage parasites. Consequently, ring-stage parasites treated with guanidinium chloride could develop into mature parasite stages following treatment, facilitating the potential use of this solution to synchronise *P. berghei* when cultured in vitro. The guanidinium lysis assay developed here fulfilled the project’s aims and is an effective tool in assessing NPP activity in a rodent malaria model.

## 2. Materials and Methods

### 2.1. Ethics Statement and Mice

Female ARC(s) Swiss mice (6–10 weeks) or Wistar rats (6 weeks) were sourced from the Animal Resource Centre (Perth, Australia). They were maintained on a standard rodent diet (chow) and housed under controlled conditions at 21 °C with a 12:12 h light:dark cycle. All experiments were performed in accordance with the recommendations of the National Health and Medical Research Council Australian ‘Code of practice for the care and use of animals for scientific purposes’ and approved by the Deakin University Animal Welfare Committee (Project G03-2020 & G03-2023).

### 2.2. Culturing of Plasmodium berghei Parasites

Stabilites of RBCs infected with the *P. berghei* ANKA strain clone 15 cy1 stored in liquid nitrogen were thawed and injected interperitoneally (IP) into a donor mouse. Experimental mice or rats were infected with 1 × 10^6^ iRBCs or 3 × 10^7^ iRBCs, respectively, which had been harvested from donor mice. The parasite load in the animals was monitored via Giemsa-stained blood smears. Cardiac bleeds were performed on the infected mice and rats when the parasitemia reached >10% or >5%, respectively; blood was also simultaneously harvested from age- and sex-matched uninfected animals to serve as controls. The collected infected blood contained ring- or trophozoite-stage parasites and was washed with incomplete RPMI-HEPES culture medium (10.41 g RPMI-1640, 2 g NaHCO_3_, 5.96 g HEPES, 5 mL Neomycin, in 1 L H_2_O) and adjusted to 10% parasitemia with uninfected blood. The samples were further cultured in vitro at 5% haematocrit at 36.5 °C in complete culture medium (RPMI-HEPES containing 25% (*v*/*v*) fetal bovine serum (FBS)) under 1% O_2_ and 5% CO_2_ in N_2_, in 30 mL culture dishes for approximately 5 h to mature parasites.

### 2.3. Synchronising P. berghei Parasites

To achieve synchronous populations of *P. berghei* parasites, schizonts were collected following overnight culture (16 h). Blood smears were stained with Giemsa and the morphology of schizonts was examined by microscopy using 100× magnification under oil to ensure >70% viable schizonts distinguishable by the segmentation of merozoites. To purify the schizonts, overnight cultures were underlaid with 336 mM Iohexol (nycodenz) in buffered medium (5 mM Tris/HCl, 150 mM KCl, 5 mM CaNa_2_EDTA) at a 1:1 ratio with ice cold mouse tonicity phosphate buffered saline (MT-PBS) and spun at 290× *g* for 20 min at 22 °C using a swing-out rotor with slow acceleration and deceleration. The schizonts were visible as a brown ring at the blood/nycodenz interface and were collected and washed with 15 mL of culture media (290× *g*, 5 min). Pelleted schizonts were resuspended in fresh culture media. The volume of media used for resuspension was dependent upon the number of mice or rats that were to receive parasites by intravenous (IV) injection. Following IV injection, the parasites were left to mature and reinvade RBCs for 4 h, after which the rodents were then humanely killed by CO_2_ administration and cardiac bleeding. Blood was resuspended in fresh complete RPMI media and contained 0–4 h synchronous ring-stage parasites.

### 2.4. Osmotic Lysis Assay

Parasite cultures were pelleted by centrifugation at 500× *g* for 5 min, then resuspended with a small volume of incomplete RPMI and divided into 50 µL aliquots. To each aliquot, 1 mL of lysis solution was added. Lysis buffers included 280 mM sorbitol, 140 mM guanidinium chloride, 140 mM L-isoleucine (note that low solubility constrained use at 140 mM conc.), 280 mM L-alanine and 280 mM D-alanine, all buffered in 20 mM HEPES at a pH of 7.4. MT-PBS and 0.15 (*w*/*v*) % saponin were used as negative and positive controls for lysis, respectively. RBCs were gently resuspended and incubated at the specified temperature for 5–20 min, depending on the assay. Following incubation, the samples were centrifuged at 10,000× *g* for 1 min and the degree of lysis was determined by measuring the amount of haemoglobin released into the supernatant. Specifically, 100 µL of supernatant was pipetted into a 96-well plate in triplicate and the absorbance read on a spectrophotometer (GloMax Promega) at 560 nm wavelength. The degree of RBC lysis was determined by subtracting the optical density (OD) of the PBS negative control from the OD value obtained with the solution of interest, and the resulting value was then divided by the OD of the saponin positive control and expressed as a percentage (i.e., ([OD solution of interest − OD PBS]/[OD saponin − OD PBS]) × 100). At least three biological replicates were performed, with the results presented as mean ± standard error of the mean (SEM). Statistical significance was determined using two-way ANOVA and corrected for multiple comparisons using Tukey’s multiple comparison test for all experiments. 

## 3. Results

### 3.1. Sorbitol Lyses Both Infected and Uninfected Rodent RBCs

**Figure 1:**
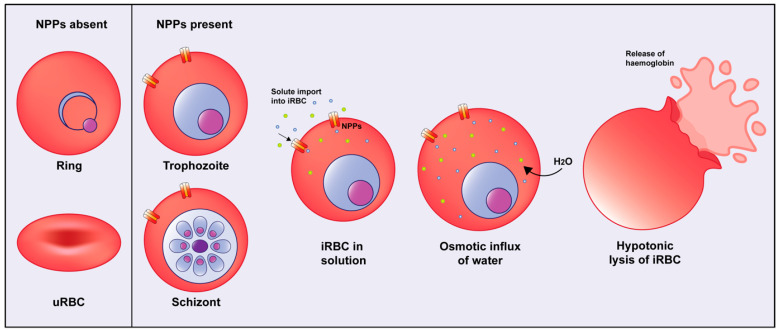
Osmotic lysis of *P. falciparum*-infected RBCs. Ring-stage parasites and uRBCs do not possess NPPs and thus remain intact following sorbitol lysis assay, as the sorbitol solution is unable to cross RBC membrane via endogenous channels. Late-stage parasites (trophozoite and schizont) possessing NPPs import an isotonic sorbitol solution into the cell, and this is accompanied by the osmotic influx of water. The iRBCs subsequently swell and burst, releasing haemoglobin and other cellular contents into the solution.

The NPP functionality in *P. falciparum* can be assessed using an isotonic sorbitol solution in combination with a measurement of haemolysis. Sorbitol cannot traverse the RBC membrane via endogenous channels but is able to cross the membrane of iRBCs, specifically via NPPs. NPPs are present at the iRBC membrane of mature trophozoite and schizont parasites but are absent from ring-stage parasites. The influx of sorbitol into iRBCs possessing NPPs causes hypotonic lysis of the cell, while uRBCs or those infected with ring-stage parasites remain intact [29] (Figure 1). Prior to being resuspended in sorbitol, the osmolarity of the iRBCs is balanced with its external environment; thus, the movement of water is balanced across the membrane. Following the import of solute via the NPPs, the osmolarity inside of the iRBCs becomes higher than that across the membrane; subsequently, water moves inside the cell by osmosis, causing lysis.

**Figure 2:**
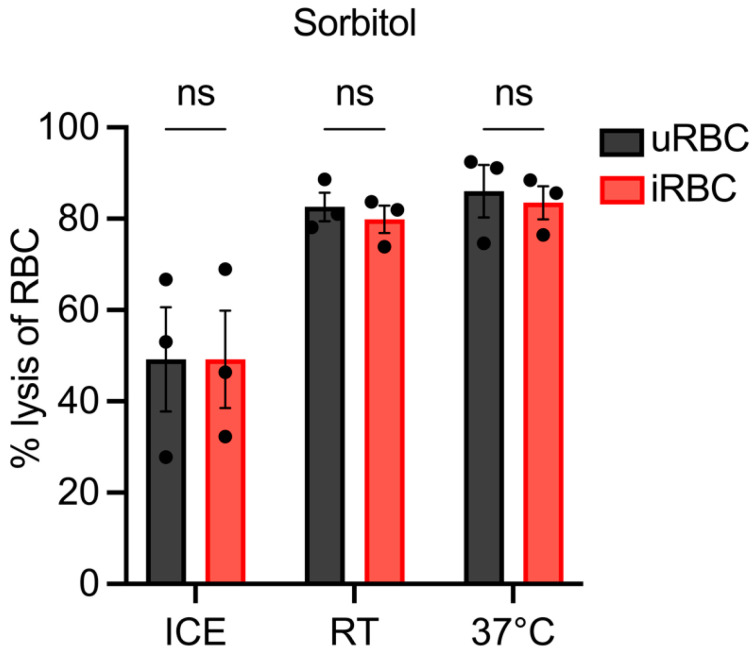
Sorbitol-mediated lysis of uninfected and *P. berghei*-infected rodent RBCs. Lysis assays (n = 3) were performed by incubating iRBCs (10% parasitemia) and uRBCs (negative control) in 280 mM sorbitol buffered in 20 mM HEPES, pH 7.4, at the indicated temperatures for 10 min. The percentage lysis was calculated relative to lysis with saponin (100% lysis). No significant (ns) difference was observed between the infected and uninfected samples at any of the indicated temperatures.

To assess NPP functionality in *P. berghei*, the first lysis reagent tested was 280 mM sorbitol, given that this is the solution most commonly used to lyse RBCs infected with *P. falciparum*. However, this concentration of sorbitol ruptured the uRBCs at all temperatures tested (Figure 2). Moreover, the degree of sorbitol-mediated lysis was not significantly different between infected and uninfected samples, with the lysis of samples ranging from 50 to 90% (Figure 2). These findings contrast with what is observed in *P. falciparum* (Ginsburg et al. 1983) and suggest that sorbitol may be transported by other endogenous transport routes. Therefore, alternate solutions were employed to determine if they could discriminately lyse RBCs infected with *P. berghei*.

### 3.2. Assessing iRBC Lysis of Isotonic Solutions at Different Temperatures

To design a haemolysis assay capable of specifically lysing *P. berghei* iRBCs, isotonic solutions known to traverse *P. falciparum* iRBCs via NPPs were analysed, including L-alanine (280 mM), D-alanine (280 mM), L-isoleucine (140 mM) and guanidinium chloride (140 mM) [5,33,34,35]. Solutions were used at these concentrations to keep them approximately isosmotic with the osmolarity of RBCs (approx. 300 mOsm/kg) [32], although in the case of L-isoleucine, the concentration was restricted on its solubility. Lysis of blood containing 10% infected cells was performed at varying temperatures (on ice, room temperature, 37 °C) to determine the optimal solution and conditions for lysis of asynchronous populations of iRBCs. It should be noted that haemoglobin concentrations in iRBCs are lower than those in uRBCs as a result of haemoglobin consumption and, therefore, values lower than 10% lysis are not necessarily indicative of incomplete lysis of a population. The percentage values in these experiments reflect a qualitative measure of lysis, rather than a quantitative measure of the proportion of iRBCs susceptible to lysis per se.

**Figure 3:**
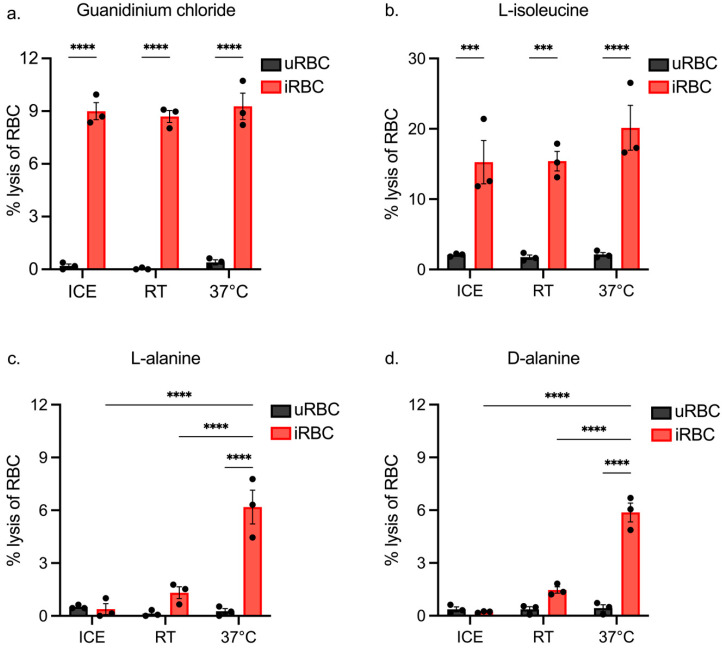
Lysis of uninfected and *P. berghei*-infected rodent RBCs using different isotonic solutions at varying temperatures. Lysis assays (n = 3) were performed on iRBCs (10% parasitemia) and uRBCs (negative control) using either guanidinium chloride (**a**), L-isoleucine (**b**), L-alanine (**c**), or D-alanine (**d**) at the specified temperatures, and the percentage of RBC lysis after 10 min was calculated relative to lysis with saponin (100% lysis) (*** *p* ≤ 0.001, **** *p* ≤ 0.0001).

The results show minimal lysis of uRBCs with guanidium chloride (Figure 3a), with on average 0.13% (±0.3%) lysis observed. Importantly, guanidinium chloride caused significantly more lysis of iRBCs compared with uRBCs, independent of temperature. L-isoleucine showed preferential lysis of iRBCs (Figure 3b), but lysis of uRBCs was higher than that of guanidium chloride. Lysis of iRBCs was on average 16.9 % (±2.8%), which is considerably higher than the expected 10% lysis, in keeping with lysis of some uRBCs. The incubation temperature again had no significant impact on the degree of lysis.

Lysis induced by both L-alanine and D-alanine was comparable (Figure 3c,d). Minimal lysis of uRBCs was observed, but lysis of iRBCs was only significantly increased at 37 °C, suggesting that uptake through NPPs is minimal at lower temperatures. Lysis induced by either isoform of alanine ranged from 5.9% to 6.2%, suggesting that iRBCs were susceptible to uptake of these isotonic solutions.

### 3.3. Assessing the Impact of Incubation Length on iRBC and uRBC Lysis by Isotonic Solutions

The optimal temperature of lysis was consistently observed to be 37 °C (Figure 3); for this reason and because it is representative of the biological temperature that parasites are exposed to in the mammalian host, this temperature was selected for all further experiments. Since the L-alanine and D-alanine results presented in Figure 3 were similar across the incubation temperatures, only guanidinium chloride, L-isoleucine and L-alanine were investigated further.

**Figure 4:**
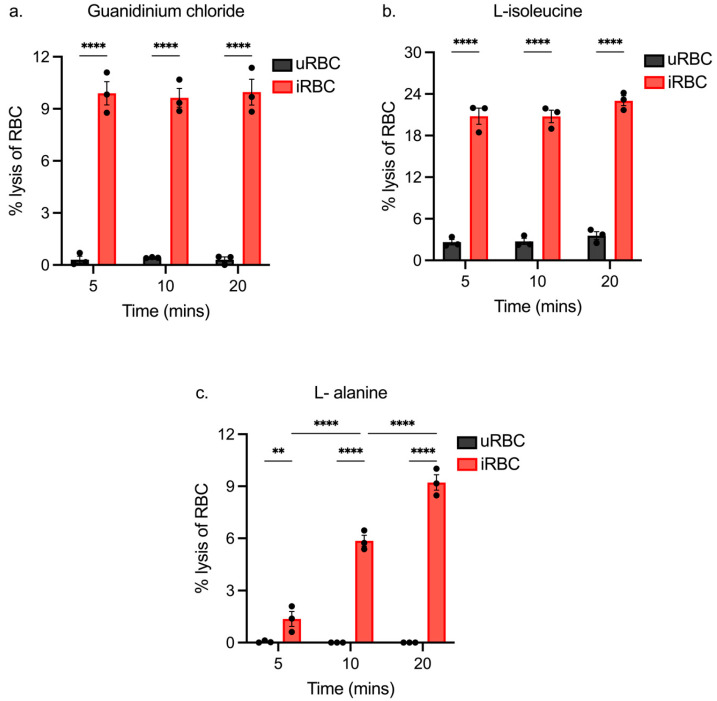
Lysis of *P. berghei*-infected and uninfected rodent RBCs at varying incubation times. Lysis assays (n = 3) were conducted on iRBC (10% parasitemia) and uRBC samples (negative control) using either guanidinium chloride (**a**), L-isoleucine (**b**), or L-alanine (**c**). The assays were carried out at 37 °C for the specified times and the percentage lysis was calculated relative to saponin (100% lysis) (** *p* ≤ 0.01, **** *p* ≤ 0.0001).

We next assessed the impact of incubation time on the degree of lysis by exposing RBCs to lysis solutions for times ranging from 5 to 20 min. Guanidinium chloride was found to cause a significant amount of iRBC lysis compared to uRBC across all incubation times (Figure 4a). On average, guanidinium chloride lysed 9.8% (±0.2%) iRBCs. uRBC lysis remained low at 0.3% (±0.07%) and did not increase with time. These results indicate that guanidinium chloride is rapidly transported into iRBCs.

The degree of iRBC lysis with L-isoleucine was also not significantly different across the three incubation times (Figure 4b), with lysis on average 21.5% (±1.3%) of RBCs. This is considerably higher than that expected for samples at 10% parasitemia and is consistent with the results observed in Figure 3b. There was a subtle increase in uRBC lysis with increased incubation time, although this increase was not statistically significant.

Extension of the incubation time significantly increased the degree of iRBC lysis with L-alanine (Figure 4c) and required 20 min to give the desired level of lysis. On average, 9.2% (±0.8%) iRBCs lysed within 20 min, which is comparable to guanidinium chloride lysis. The significant amount of time needed to achieve lysis with L-alanine indicates a slow uptake of L-alanine through NPPs.

The results thus far indicate that guanidium chloride is the optimal reagent for lysis assays, exhibiting rapid preferential lysis of iRBCs in all conditions tested. L-alanine was able to preferentially lyse iRBCs, although more slowly in comparison to guanidinium chloride. As L-isoleucine at this concentration caused excessive lysis of iRBC and uRBC samples, it was decided to move forward with guanidinium chloride and L-alanine.

### 3.4. Using NPP Inhibitors to Determine the Pathway of iRBC Lysis

Data presented in Figure 3 and Figure 4 showed that guanidinium chloride and L-alanine could preferentially lyse iRBCs, but it remained to be shown whether these solutions enter iRBCs via NPPs. Previous studies on *P. falciparum* and other rodent species of *Plasmodium* have utilised the NPP inhibitors furosemide and 5-nitro-2-(3-phenylpropylamino) benzoic acid (NPPB), which block sorbitol from entering NPPs and inhibit osmotically induced lysis of iRBCs [5,27,28]. Despite evolutionary separation, NPPs have been shown to be highly conserved across various *Plasmodium* species, including the blocking capabilities of NPP inhibitors [27,28,36]. Given that the rodent species are closely related and that the coding region of *P. falciparum clag3.1/3.2* and their *P. berghei* orthologue are reasonably well conserved, it was predicted that both furosemide and NPPB would also inhibit the *P. berghei* NPP. Thus, these inhibitors were next investigated in combination with guanidinium chloride or L-alanine to determine if lysis could be inhibited using the optimal conditions established (37 °C, 10 min). Incubations were limited to 10 min to avoid any off-target effects potentially caused by inhibitors.

**Figure 5:**
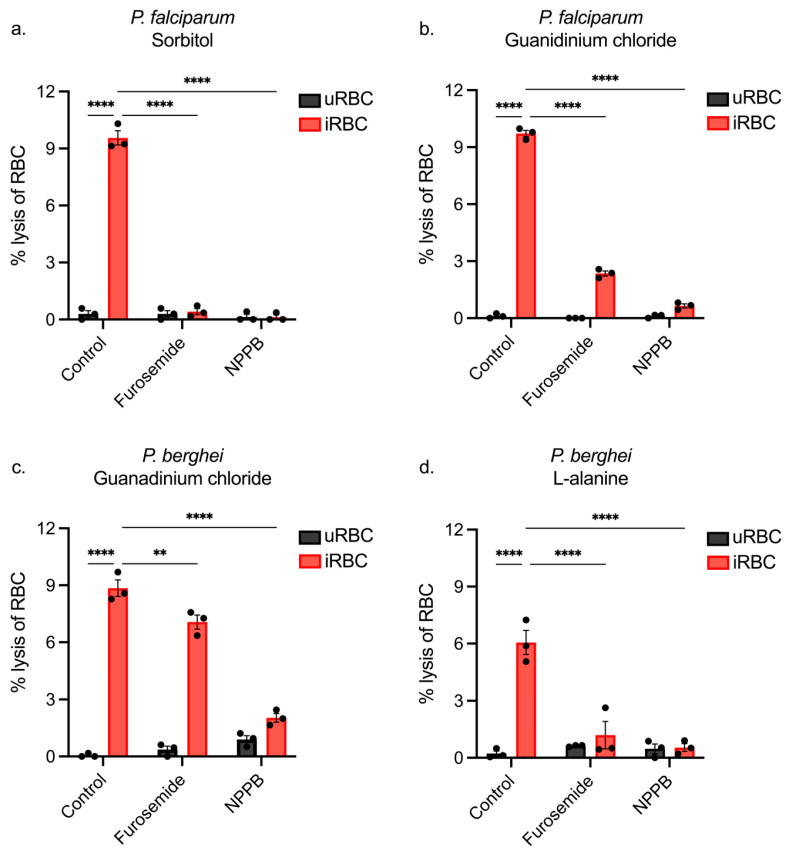
Lysis of *P. berghei*- and *P. falciparum*-infected and uninfected RBCs using NPP inhibitors furosemide and NPPB. Lysis assays (n = 3) were conducted on iRBCs (10% parasitemia) and uRBCs (negative control) from *P. falciparum* (**a**,**b**) and *P. berghei* (**c**,**d**) using either sorbitol (**a**), guanidinium chloride (**b**,**c**), or L-alanine (**d**). The assays were carried out at 37 °C for 10 min in the presence and absence of 200 μM furosemide, 200 μM 5-nitro-2-(3-phenylpropylamino) benzoic acid (NPPB) or DMSO as a negative (vehicle) control, and the percentage lysis was calculated relative to saponin (100% lysis) (** *p* ≤ 0.01, **** *p* ≤ 0.0001).

To validate the inhibitor results, *P. falciparum* cultures were treated with each of these inhibitors to observe the effect on sorbitol-induced lysis. Furosemide and NPPB were both potent inhibitors of lysis of *P. falciparum* iRBCs, demonstrating that sorbitol effectively lyses the RBCs directly via NPPs (Figure 5a). Guanidinium lysis assays were performed on *P. falciparum* cultures, and there was a significant reduction in iRBC lysis with both furosemide and NPPB treatment; however, it was not completely inhibited using furosemide (75.82% inhibition) (Figure 5b). Nevertheless, lysis of uRBCs was near zero at 0.04% (±0.24%), meaning that a parasite-induced channel in the human RBC likely transports guanidinium chloride into the iRBC. Treatment of *P. berghei* iRBCs with furosemide significantly decreased guanidinium chloride-induced lysis, but again, the inhibition was not complete (20.15% inhibition). In comparison, the addition of NPPB prevented the lysis of *P. berghei* iRBCs by guanidinium chloride to an average degree of 77.01% inhibition (Figure 5c). Interestingly, both furosemide and NPPB were capable of inhibiting L-alanine-induced lysis, decreasing it to 1.2% (±1.2%) and 0.54% (±0.4%), respectively, such that there was no significant difference in lysis to uRBCs. These results indicate that the NPP structure must be sufficiently conserved between *P. falciparum* and *P. berghei* to facilitate the binding and blocking of the channel. The lack of complete blocking of the NPP by furosemide using guanidinium chloride in either species likely stems from the rapid uptake of guanidinium into the iRBC.

### 3.5. Lysis of Synchronous Populations of P. berghei iRBCs

To this point, the parasites utilised in the lysis experiments were from asynchronous cultures. Thus, the degree of lysis could have been reduced because some parasites may have been too early in their development to express NPPs. Due to the lack of previous studies, it is unknown precisely when NPPs are formed in rodent malaria parasites and their shorter lifecycle (~24 h) may influence their establishment. To explore this further, lysis assays were carried out on synchronous populations of ring and trophozoite parasites to determine if the parasite stage impacts NPP activity.

For these experiments, synchronous populations of ring-stage parasites at 0–4 h post-invasion at an average parasitemia of 1.65% (±0.32%) were collected from the infected rodents and an osmotic lysis assay was performed on half the material. The remaining material was cultured until the parasites reached the trophozoite stage (~12 h post-invasion), and then the lysis assay was repeated using guanidinium chloride, selected on the basis that this solute gave the most robust results with osmotic lysis assays. Both assays were carried out under identical conditions, with an incubation temperature and time of 37 °C and 10 min.

**Figure 6:**
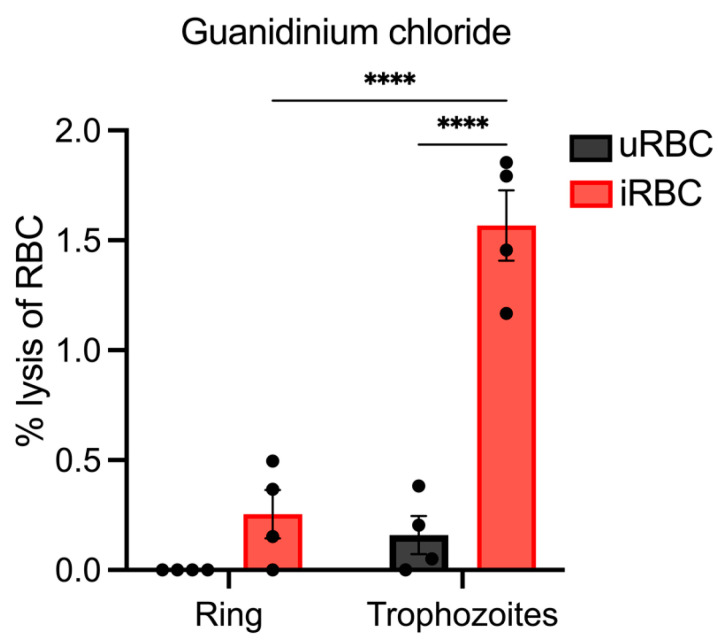
Lysis of synchronous populations of *P. berghei*-infected and uninfected RBC. Lysis assays (n = 4) were conducted on iRBC (average parasitemia 1.65%, ±0.32%) and uRBC samples (negative control) using guanidinium chloride. The assays were carried out at 37 °C for 10 min and the percentage lysis was calculated relative to saponin (100% lysis). Populations of ring and trophozoite parasites were used in the assay, where trophozoites were matured from ring stages in vitro (**** *p* ≤ 0.0001).

Lysis of *P. berghei* trophozoite populations was significantly higher than ring-stage parasites, averaging 1.57% (±0.32%) and 0.25% (±0.22%), respectively (Figure 6), and significantly higher than lysis of uRBCs. This indicates that most trophozoite iRBCs had lysed. Conversely, ring-stage parasites were not significantly lysed compared to uRBCs. These results suggest that not only is guanidinium chloride lysis facilitated by uptake via NPPs, but that NPPs are also likely expressed during the same stages of *P. berghei* development as in *P. falciparum* during the trophozoite stage. 

### 3.6. P. berghei Survival and Growth following Guanidinium Lysis Assay

At this stage, guanidinium chloride has been shown to effectively cause lysis of trophozoite *P. berghei* iRBCs possessing NPPs and is, therefore, an effective tool for determining NPP functionality. Whether or not the assay negatively affected the health of parasites and whether it could also be used to synchronise populations of parasites remained to be shown. Therefore, parasite growth and development were monitored following the synchronisation of a population of parasites. 

**Figure 7:**
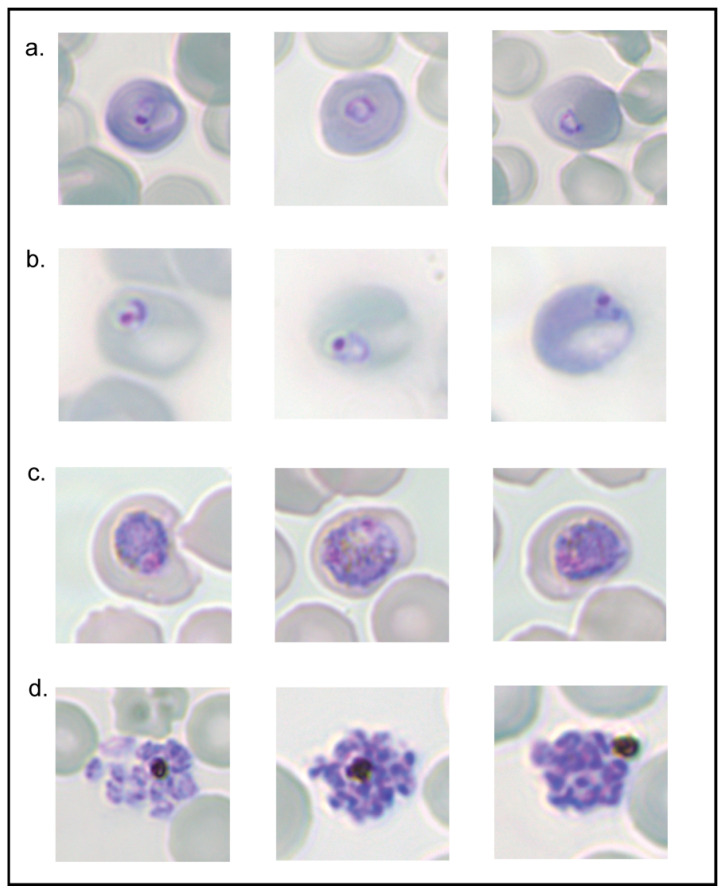
Survival assay following guanidinium chloride lysis assay. (**a**) Ring-stage *P. berghei* parasites 0-4 h prior to resuspension in guanidinium chloride. (**b**) Ring-stage parasites following resuspension in guanidinium chloride. (**c**) Trophozoite-stage parasites ~16 h later. (**d**) Schizont-stage parasites ~24 h following guanidinium chloride lysis assay (n = 3).

Parasites were collected from the infected mice, cultured to schizonts, and purified using Nycodenz solution. The schizonts were then injected IV into the mice and allowed to reinvade for ~4 h. Subsequently, the mice were cardiac bled, and a population of ring-stage parasites between 0 and 4 h was collected. The parasites were collected in this way to ensure the presence of early ring-stage parasites not possessing NPPs that would survive resuspension in guanidinium solution. From each mouse, 500 µL of blood was resuspended in 5 mL of pre-warmed guanidinium chloride and incubated at 37 °C for 10 min. Blood was then pelleted, washed three times and then cultured for ~24 h. Smears of parasite growth before and following incubation in guanidinium chloride at intervals are represented in Figure 7.

Ring-stage parasites were shown to be present before and after resuspension in guanidinium chloride. Importantly, ~16 h following this, iRBCs were smeared and the parasites had progressed to the late trophozoite stage. At the end of the 24 h incubation, the parasites were able to progress to the schizont stage. This indicates that treatment of ring-stage parasites in guanidinium chloride still enables their progression into mature parasites. 

## 4. Discussion

NPPs facilitate nutrient import into iRBCs and have been shown to be essential for the *P. falciparum* lifecycle. This study aimed to establish an NPP assay for *P. berghei* by testing four different osmotic solutions for their ability to preferentially lyse iRBCs and the impact of time and temperature on their effectiveness.

This study showed that guanidinium chloride was able to specifically enter the parasitised rodent RBC via NPPs and cause hypotonic lysis of the cell. Changes to assay incubation temperatures and times showed that guanidinium chloride was significantly effective across all three temperatures (ice, RT, 37 °C) and was rapidly taken up by iRBCs, since significant lysis was already observed within 5 min. Importantly, uRBCs were not significantly lysed at any given temperature, and lysis did not increase with time. In comparison, L-isoleucine was also able to induce a significant amount of lysis of iRBCs across the range of temperatures and times tested; however, the amount of lysis was higher than that expected for the given parasitemia, indicating that it also causes lysis of uRBCs. This is not unexpected, since the concentration of L-isoleucine used was constrained by its solubility and was likely hypo-osmotic. D-alanine and L-alanine gave a similar degree of RBC lysis; notably, there was little, if any, lysis of uRBCs across the range of temperatures and times tested in the assay, signifying that it was not being taken up or having a lysis effect on uRBCs. Given that lysis of iRBCs was only significant at 37 °C and significantly increased with an extended incubation time of 20 min, this indicates that the uptake of D-alanine and L-alanine by NPPs is significantly slower than the uptake of guanidinium chloride.

Blocking the NPPs with furosemide and NPPB revealed that guanidinium chloride-induced lysis of *P. berghei* iRBCs was significantly reduced with furosemide and almost completely inhibited by NPPB. Both inhibitors blocked L-alanine-induced lysis such that lysis of iRBCs was not significantly different than lysis of uRBCs. The inability of furosemide to fully inhibit guanidinium-induced lysis of *P. berghei* iRBCs indicated that furosemide binds less well to the rodent malaria NPP. However, furosemide has been shown to be a potent inhibitor of L-adenosine influx (IC_50_ 15 μM) in RBCs infected with the rodent species *P. yoelli* [28]. Moreover 100 μM of furosemide or NPPB could inhibit choline influx in RBCs infected with *P. falciparum* and protect against haemolysis [5]. Therefore, it is likely that our results signify that the flux of guanidinium chloride across the channel occurred more rapidly than the time taken to effectively block the channel with furosemide, given that it was applied in the lysis solution. The almost complete inhibition of NPP activity by NPPB reflected a higher binding affinity to the NPP of *P. berghei* parasites than furosemide, given the almost complete inhibition of lysis seen.

The use of synchronised populations of *P. berghei* provided further evidence that the pathway of guanidinium lysis is via NPPs and was consistent with the establishment of NPPs during the trophozoite stage. Lysis of earlier ring stages compared to matured trophozoites was significantly reduced, suggesting that *P. berghei* ring stages lack the channel capable of importing guanidinium. This may be because of the time taken to traffic and establish the NPP constituents at the iRBC membrane. Given that NPPs in *P. falciparum* also do not establish until the trophozoite stage [37,38], this provides further support that NPPs are the pathway of guanidinium-induced lysis in *P. berghei* iRBCs.

Given the lethal effect that guanidinium chloride had on mature parasites, it was important to show that ring-infected RBCs exposed to the solution would not be negatively impacted for the remainder of their lifecycle. Washing the blood pellet was an extremely important step to prevent any guanidinium chloride remaining in the culture media from being imported into iRBCs as the parasites matured. Our results suggest that parasites were able to grow and progress to the schizont stage, undergoing segmentation and generating merozoites. The guanidinium assay could, therefore, not only be used to determine NPP functionality, but possibly to synchronise populations of parasites. Currently, attaining a population of synchronous ring-stage parasites requires multiple stages and days of infections and culturing as well as intravenous infections and nycodenz purification. Using this alternative method on a mixed population of parasites could allow the synchronisation of parasites in a single step and in a significantly shorter period of time.

The assay may potentially be improved further by balancing the osmolarity of buffered guanidinium chloride solution more precisely to the slightly higher osmolarity of the mouse RBC (327 mOsm/kg ± 14.57) [32]. However, given that the assay does not induce a significant amount of uRBC lysis, this may very well be unnecessary. Furthermore, if the assay is to be used to synchronise populations of parasites, the toxicity of guanidinium chloride to ring-stage parasites should be further investigated in a quantitative manner.

## 5. Conclusions

The guanidinium chloride osmotic lysis assay designed in this study is robust enough to assess the functionality of *P. berghei* NPPs. Furthermore, it could theoretically be used to synchronise populations of mixed-stage parasites in a way analogous to how sorbitol is used to synchronise populations of *P. falciparum* parasites, given the survival of ring-stage parasites and progression to the schizont stage. Further studies of the genetic contribution of *clag* genes to rodent malaria *P. berghei* NPPs should now be explored using a guanidinium lysis assay to better understand the molecular structure of the essential channel.

## Data Availability

Data available on request.

## References

[1] Straimer J., Gnädig N.F., Witkowski B., Amaratunga C., Duru V., Ramadani A.P., Dacheux M., Khim N., Zhang L., Lam S. (2015). K13-propeller mutations confer artemisinin resistance in *Plasmodium falciparum* clinical isolates. Science.

[2] Uwimana A., Legrand E., Stokes B.H., Ndikumana J.-L.M., Warsame M., Umulisa N., Ngamije D., Munyaneza T., Mazarati J.-B., Munguti K. (2020). Emergence and clonal expansion of in vitro artemisinin-resistant Plasmodium falciparum kelch13 R561H mutant parasites in Rwanda. Nat. Med..

[3] Uwimana A., Umulisa N., Venkatesan M., Svigel S.S., Zhou Z., Munyaneza T., Habimana R.M., Rucogoza A., Moriarty L.F., Sandford R. (2021). Association of Plasmodium falciparum kelch13 R561H genotypes with delayed parasite clearance in Rwanda: An open-label, single-arm, multicentre, therapeutic efficacy study. Lancet Infect. Dis..

[4] Desai S.A., Krogstad D.J., McCleskey E.W. (1993). A nutrient-permeable channel on the intraerythrocytic malaria parasite. Nature.

[5] Kirk K., Horner H., Elford B., Ellory J., Newbold C. (1994). Transport of diverse substrates into malaria-infected erythrocytes via a pathway showing functional characteristics of a chloride channel. J. Biol. Chem..

[6] Pillai A.D., Pain M., Solomon T., Bokhari A.A.B., Desai S.A. (2010). A Cell-Based High-Throughput Screen Validates the Plasmodial Surface Anion Channel As an Antimalarial Target. Mol. Pharmacol..

[7] Overman R.R. (1948). Reversible cellular permeability alterations in disease; in vivo studies on sodium, potassium and chloride concentrations in erythrocytes of the malarious monkey. Am. J. Physiol..

[8] Ginsburg H., Krugliak M., Eidelman O., Cabantchik Z.I. (1983). New permeability pathways induced in membranes of Plasmodium falciparum infected erythrocytes. Mol. Biochem. Parasitol..

[9] Desai S.A., Bezrukov S.M., Zimmerberg J. (2000). A voltage-dependent channel involved in nutrient uptake by red blood cells infected with the malaria parasite. Nature.

[10] Staines H.M., Alkhalil A., Allen R.J., De Jonge H.R., Derbyshire E., Egée S., Ginsburg H., Hill D.A., Huber S.M., Kirk K. (2007). Electrophysiological studies of malaria parasite-infected erythrocytes: Current status. Int. J. Parasitol..

[11] Nguitragool W., Bokhari A.A., Pillai A.D., Rayavara K., Sharma P., Turpin B., Aravind L., Desai S.A. (2011). Malaria Parasite clag3 Genes Determine Channel-Mediated Nutrient Uptake by Infected Red Blood Cells. Cell.

[12] Nguitragool W., Rayavara K., Desai S.A. (2014). Proteolysis at a Specific Extracellular Residue Implicates Integral Membrane CLAG3 in Malaria Parasite Nutrient Channels. PLoS ONE.

[13] Gupta A., Bokhari A.A.B., Pillai A.D., Crater A.K., Gezelle J., Saggu G., Nasamu A.S., Ganesan S.M., Niles J.C., Desai S.A. (2020). Complex nutrient channel phenotypes despite Mendelian inheritance in a Plasmodium falciparum genetic cross. PLoS Pathog..

[14] Comeaux C.A., Coleman B.I., Bei A.K., Whitehurst N., Duraisingh M.T. (2011). Functional analysis of epigenetic regulation of tandem RhopH1/clag genes reveals a role in Plasmodium falciparum growth. Mol. Microbiol..

[15] Pillai A.D., Nguitragool W., Lyko B., Dolinta K., Butler M.M., Nguyen S.T., Peet N.P., Bowlin T.L., Desai S.A. (2012). Solute restriction reveals an essential role for clag3-associated channels in malaria parasite nutrient acquisition. Mol. Pharmacol..

[16] Counihan N.A., Chisholm S.A., Bullen H.E., Srivastava A., Sanders P.R., Jonsdottir T.K., Weiss G.E., Ghosh S., Crabb B.S., Creek D.J. (2017). Plasmodium falciparum parasites deploy RhopH2 into the host erythrocyte to obtain nutrients, grow and replicate. eLife.

[17] Ito D., Schureck M.A., Desai S.A. (2017). An essential dual-function complex mediates erythrocyte invasion and channel-mediated nutrient uptake in malaria parasites. eLife.

[18] Sherling E.S., Knuepfer E., Brzostowski J.A., Miller L.H., Blackman M.J., van Ooij C. (2017). The Plasmodium falciparum rhoptry protein RhopH3 plays essential roles in host cell invasion and nutrient uptake. eLife.

[19] Ho C.M., Li X., Lai M., Terwilliger T.C., Beck J.R., Wohlschlegel J., Goldberg D.E., Fitzpatrick A.W.P., Zhou Z.H. (2020). Bottom-up structural proteomics: CryoEM of protein complexes enriched from the cellular milieu. Nat. Meth..

[20] Schureck M.A., Darling J.E., Merk A., Shao J., Daggupati G., Srinivasan P., Olinares P.D.B., Rout M.P., Chait B.T., Wollenberg K. (2021). Malaria parasites use a soluble RhopH complex for erythrocyte invasion and an integral form for nutrient uptake. eLife.

[21] Gupta A., Thiruvengadam G., Desai S.A. (2015). The conserved clag multigene family of malaria parasites: Essential roles in host–pathogen interaction. Drug Resist. Updat..

[22] Iriko H., Kaneko O., Otsuki H., Tsuboi T., Su X.-z., Tanabe K., Torii M. (2008). Diversity and evolution of the rhoph1/clag multigene family of Plasmodium falciparum. Mol. Bioch. Parasitolo..

[23] Janse C.J., Ramesar J., Waters A.P. (2006). High-efficiency transfection and drug selection of genetically transformed blood stages of the rodent malaria parasite *Plasmodium berghei*. Nat. Protoc..

[24] Tokunaga N., Nozaki M., Tachibana M., Baba M., Matsuoka K., Tsuboi T., Torii M., Ishino T. (2019). Expression and Localization Profiles of Rhoptry Proteins in *Plasmodium berghei* Sporozoites. Front. Cell. Infect. Microbiol..

[25] Homewood C.A., Neame K.D. (1974). Malaria and the permeability of the host erythrocyte. Nature.

[26] Huber S.M., Duranton C., Henke G., van de Sand C., Heussler V., Shumilina E., Sandu C.D., Tanneur V., Brand V., Kasinathan R.S. (2004). Plasmodium Induces Swelling-activated ClC-2 Anion Channels in the Host Erythrocyte. J. Biol. Chem..

[27] Staines H.M., Kirk K. (1998). Increased choline transport in erythrocytes from mice infected with the malaria parasite Plasmodium vinckei vinckei. Biochem. J..

[28] Gati W.P., Lin A.N., Wang T.I., Young J.D., Paterson A.R. (1990). Parasite-induced processes for adenosine permeation in mouse erythrocytes infected with the malarial parasite Plasmodium yoelii. Biochem. J..

[29] Lambros C., Vanderberg J.P. (1979). Synchronization of Plasmodium falciparum Erythrocytic Stages in Culture. J. Parasitol..

[30] An X., Schulz V.P., Mohandas N., Gallagher P.G. (2015). Human and murine erythropoiesis. Curr. Opin. Hematol..

[31] Pasini E.M., Kirkegaard M., Salerno D., Mortensen P., Mann M., Thomas A.W. (2008). Deep Coverage Mouse Red Blood Cell Proteome. Mol. Cell. Proteom..

[32] Varga A., Matrai A.A., Barath B., Deak A., Horvath L., Nemeth N. (2022). Interspecies Diversity of Osmotic Gradient Deformability of Red Blood Cells in Human and Seven Vertebrate Animal Species. Cells.

[33] Bokhari A.A.B., Mita-Mendoza N.K., Fuller A., Pillai A.D., Desai S.A. (2014). High Guanidinium Permeability Reveals Dehydration-Dependent Ion Selectivity in the Plasmodial Surface Anion Channel. BioMed Res. Int..

[34] Martin R.E., Kirk K. (2007). Transport of the essential nutrient isoleucine in human erythrocytes infected with the malaria parasite Plasmodium falciparum. Blood.

[35] Ngernna S., Chim-Ong A., Roobsoong W., Sattabongkot J., Cui L., Nguitragool W. (2019). Efficient synchronization of Plasmodium knowlesi in vitro cultures using guanidine hydrochloride. Malar. J..

[36] Lisk G., Desai S.A. (2005). The plasmodial surface anion channel is functionally conserved in divergent malaria parasites. Eukaryot. Cell.

[37] Ginsburg H., Kutner S., Krugliak M., Cabantchik Z.I. (1985). Characterization of permeation pathways appearing in the host membrane of Plasmodium falciparum infected red blood cells. Mol. Biochem. Parasitol..

[38] Staines H.M., Ellory J.C., Kirk K. (2001). Perturbation of the pump-leak balance for Na(+) and K(+) in malaria-infected erythrocytes. Am. J. Physiol. Cell Physiol..

